# Triiodothyronine potentiates angiogenesis-related factor expression through PI3K/AKT signaling pathway in human osteoarthritic osteoblasts 

**DOI:** 10.22038/ijbms.2020.43634.10252

**Published:** 2020-06

**Authors:** Lei Li, Yiqun Pang, Linlin Zhang, Meng Li, Chen Zhu, Shiyuan Fang, Zongsheng Yin

**Affiliations:** 1 Department of Orthopaedics,the First Affiliated Hospital of University of Science and Technology of China, #17 Lujiang Road, Hefei, Anhui, China; 2 Department of Orthopaedics, the First Affiliated Hospital of Anhui Medical University, #269 Jixi Road, Hefei, Anhui, China; 3 Department of Radiology, the First Affiliated Hospital of University of Science and Technology of China, #17 Lujiang Road, Hefei, Anhui, China

**Keywords:** HIF-1α, Osteoarthritis, Osteoblast, PI3K, Thyroid hormone, VEGF

## Abstract

**Objective(s):**

Previous study has indicated that triiodothyronine (T3) facilitated cartilage degeneration in osteoarthritis (OA). This study aimed to investigate the effects of T3 on angiogenesis-related factor expression in human osteoblasts of OA subchondral bone.

**Materials and Methods:**

The subchondral bone specimens were obtained from OA patients and healthy participants. The expressions of VEGF, HIF-1α, AKT, and phosphorylated AKT was detected by immunohistochemistry, Western blotting, and RT-qPCR in OA. Angiogenesis-related factor expression in OA osteoblasts was measured by treating different concentrations of T3. The hypoxia model and PX-478 (HIF-1α inhibitor) were employed to confirm the regulative role of HIF-1α for VEGF expression. The level of VEGF secretion was examined in osteoblasts supernatant using ELISA.

**Results:**

Immunohistochemistry showed strong staining of VEGF and HIF-1α in OA subchondral bone. The expression of VEGF, HIF-1α, and p-AKT in OA osteoblasts was higher than that of normal osteoblasts at protein and mRNA levels. The physiological concentration of T3 (10-7 M) in OA osteoblasts up-regulated the expression of VEGF, HIF-1α, and p-AKT after 24 hr and 48 hr culture, while a higher dose of T3 displayed the adverse effects. Additionally, VEGF and p-AKT expression was down-regulated when PX-478 inhibited HIF-1α protein.

**Conclusion:**

Our results suggested that local T3 could effectively increase angiogenesis-related factor expression by PI3K/AKT signaling pathway, and HIF-1α regulated the VEGF expression in OA osteoblasts.

## Introduction

Osteoarthritis is a degenerative disease of extremely high incidence in the elderly, which is primarily treated by pain management and surgical intervention to attenuate the joint dysfunction (1). Growing evidence has indicated that OA is not a simple process of articular cartilage degradation but an organ disorder, including synovitis, abnormal remodeling of subchondral bone and osteophyte formation (2-4). However, numerous chemokines, cytokines, and metalloproteinases have been identified as vital pathogenic factors for the onset and progression in OA subchondral bone. As a typical pathological process of OA progression, the crosstalk of bone and cartilage allows the transport of inflammatory factors and small molecules in the osteochondral unit, which further contributes to cartilage degradation (5, 6). 

Angiogenesis is considered to rely on a delicate balance between endogenous stimulators and inhibitors in subchondral bone (7). Various mediators contribute to the angiogenic process, such as VEGF, insulin-like growth factor (IGF)-1 and transforming growth factor (TGF)-β. Notably, VEGF secreted from OA osteoblasts plays a critical role in migration of vascular endothelial progenitor cells in osteochondral junction and inhibits the activation of chondrocytes (8). However, ambiguous mechanisms of microangiogenesis in subchondral bone have become the research focus and potential therapeutic targets in ameliorating OA development. Meanwhile, articular cartilage is an avascular tissue (9). Its nutrition and oxygen gradient is supplied by diffusion from the synovial fluid and subchondral bone. Grimshaw *et al*. (10) demonstrated that O_2_ concentration across thin and erosional cartilage may be altered during OA progression. HIF-1α has been reported by multiple studies concerned with cartilage degradation (11-13). As a nuclear transcription factor, HIF-1α binds to the consensus sequence of the VEGF promoter, thereby forming the “hypoxia response element” to induce angiogenesis (14).

Triiodothyronine (T3) has as an important role in bone remodeling and up-regulates genes expression of angiogenesis-related factors, such as VEGF, PGF (placental growth factor), and IGFs in multiple tissues (15, 16). Additionally, Moeller *et al*. (17) also confirmed that HIF-1α is one of the target genes of T3 via the analysis of cDNA microarray in human fibroblasts. Therefore, the present study aimed to explore whether T3 potentiated the expression of angiogenesis-related factors in OA osteoblasts and whether HIF-1α regulated VEGF production. The PI3K/AKT signaling pathway was also investigated in this process. 

## Materials and Methods

***Reagents***

DMEM/F12 medium and 1% penicillin-streptomycin mixture were purchased from Hyclone (Logan, Utah, USA). Fetal bovine serum (FBS) was procured from CLARK (CLARK Bioscience, Australia). T3, type I and II collagenases were supplied by Sigma (St Louis, MO, USA) and PX-478 was bought from MCE (Shanghai, China). Anti-VEGF, AKT, and phosphorylated AKT antibodies were obtained from Elabscience Biotechnology Co.Ltd (Wuhan, China). The anti-hif-1α antibody was bought from Abcam (Cambridge, UK). ELISA kit was obtained from the Dakewe company (Dakewe Biotech, Shenzhen, China). The RNA reverse transcription kit and quantitative polymerase chain reaction (PCR) system were purchased from TaKaRa (TaKaRa Bio, Japan).

***Extraction and culture of human osteoarthritic osteoblasts (OB)***

A total of 12 patients (3 males, 9 females, mean age 70.33±7.9 years) with OA and 5 healthy individuals (2 male, 3 females, mean age 66.8±8.17 years) were recruited in this study. The diagnose of osteoarthritis was based on the American College of Rheumatology clinical criteria (18). Tibial plateaus were obtained from OA patients undergoing total knee replacement and healthy specimens from amputees. According to Kellgren-Lawrence (KL) grade, all OA bone samples were defined as KL grade 4. None of the participants had a history of bone metabolic diseases or hormone use. The demographic parameters of all participants were listed in Table 1. The study was carried out in the first affiliated hospital of Anhui medical university and obtained approval from the ethics committee according to the Helsinki Declaration. 

 Isolation of subchondral bone and osteoblasts culture was performed as previously described (19). Moreover, osteoblasts from sclerotic (SC-OB) and non-sclerotic (NSC-OB) regions within the same joint were cultured separately. Briefly, bone samples with removed marrow tissue were excised into small chips of approximately 1~2 mm^2^ prior to the sequential digestion in the 1 mg/ml type I collagenase in DMEM/F12 medium without serum at 37 ^°^C for 20, 20, and 240 min. Bone pieces were cultured in DMEM/F12 medium containing 20% FBS. This medium was replaced every 7 days until cells were observed in the T25cm^2 ^flasks, at which time the medium within 10% FBS was changed twice a week. The osteoblasts were divided into passages at 70–80% confluence using 0.05% trypsin/0.53 mM EDTA (Beyotime, Shanghai, China). Similarly, osteoblasts from healthy specimens were cultured and served as the control group. The cells were seeded at a density of 1 × 10^6 ^cells/well in a 6-well plate for Western blotting and total RNA extraction. 

***Cell viability detection***

The viability of osteoblasts was detected by cell counting kit-8 assay (CCK-8, Beyotime, Shanghai, China) after PX-478 and hypoxia treatment. Briefly, 10 μl of CCK-8 solution was added to the culture medium in a 96-well plate and further incubated for 2–4 hr. The absorbance at 450 nm was measured by a microplate reader (Thermo Scientific, USA).

***Immunohistochemistry***

The human specimens of subchondral bone (sclerotic, non-sclerotic and normal regions) were soaked with 4% paraformaldehyde, decalcified with 10% ethylenediaminetetraacetic acid (EDTA) for one month and embedded in paraffin. Slides of approximately 5 µm thickness were deparaffinized in xylene three times and subsequently rehydrated through gradient concentrations of ethanol. Slides were rinsed out and incubated in sodium citrate solution buffer at 92 ^°^C for 10 min to perform antigen retrieval. 0.3% hydrogen peroxide was dropwised in tissue sections and incubated for 20 min at room temperature. Then, slides were incubated with anti-VEGF antibody (E-AB-40004, Elabscience) at a dilution of 1:100 and anti-HIF-1α antibody (ab51608, Abcam) at a dilution of 1:100. As secondary antibody, HRP antirabbit IgG (G23303, Servicebio, Wuhan, China) at a dilution of 1: 200 was used. Each slide was washed with PBS and stained using DAB reagent (G1211, Servicebio, Wuhan, China). Each slide immunostained for HIF-1α or VEGF was observed and scanned with a light microscope (Olympus, Japan). The percentage of positive-stained cells in total number of osteoblasts was shown by Image J software.

***Real-time quantitative PCR***

Total RNA was extracted using TRIzol (Invitrogen, USA) in OA osteoblasts. According to the manufacturer’s instructions, cDNA was synthesized through reverse transcription using the PrimeScript™RT reagent Kit with total RNA as template. Primers sequence of genes (Human): VEGF: Forward: 5’-AGGGCAGAATCATCACGAAGT-3’, Reverse: 5’-AGGGTCTCGATTGGATGGCA-3’;HIF-1α: Forward: 5’-TGATTGCATCTCCATCTCCTACC-3’, Reverse: 

5’-GACTCAAAGCGACAGATAACACG-3’; Actin: Forward: 5’-CACCCAGCACAATGAAGATCAAGAT-3’, Reverse: 5’-

CCAGTTTTTAAATCCTGAGTCAAGC-3’. 

Quantitative PCR reaction conditions were: 95 ^°^C for 30 sec, followed by 40 cycles of 95 ^°^C for 3 sec and 60 ^°^C for 30 sec. Ct (cycle threshold) values were calculated using the 2^-ΔΔCT^ method and quantification of genes expression was normalized to endogenous reference actin.

***Western blotting***

The total protein of osteoblasts was extracted by RIPA (cell lysis solutions, Beyotime, Shanghai, China), and bicinchoninic acid protein assay was used to measure protein concentration. The 10 % SDS-PAGE gel was prepared to perform electrophoresis with 20 µg protein from each sample based on standard condition, and then proteins were transferred to the 0.45 µm PVDF membrane and blocked with 5% skimmed milk for 2 hr at room temperature. Next, primary antibodies including anti-VEGF (1: 1000, E-AB-40004, Elabscience), anti-HIF-1α (1:1000, ab51608, Abcam), anti-AKT (1:1000, E-AB-30467, Elabscience), anti-phosphor-AKT (1:1000, E-AB-21187, Elabscience), and anti-GAPDH (1:10000, ab181602, Abcam) or anti-β-actin antibody (1:10000, 20536-1-AP, proteintech, China) were incubated at 4 °C overnight. The next day, protein bands were visualized by ECL luminescent liquid after incubation of secondary antibody labeled HRP (1:10000, BA1056, BOSTER, Wuhan, China) for 2 hr. The expression level of each protein was captured by Image J software and was normalized to endogenous control GAPDH or β-actin.

***Hypoxic model***

The osteoblasts were cultured in the hypoxia device containing 95% N_2_ and 5% CO_2_ for 24 hr with or without T3, and then the expression of angiogenesis-related factors was detected by Western blotting and RT-qPCR.

***Enzyme-linked immunosorbent assay***

The levels of VEGF secretion in the osteoblasts supernatant were detected using an ELISA kit, following the manufacturer’s instructions. Absorption at 450 nm was measured by a microplate reader, and quantitation of VEGF was determined according to the standard curve. The sensitivity of the assay was 1 pg/ml.

***Statistical analysis***

All experiments and treatments for cells were repeated at least three times. Data were represented as mean±SD. Comparison of different groups was performed by one-way ANOVA, followed by the Student-Newmann-Keuls *post hoc *test to determine the difference among multiple groups. The critical value of statistical significance was *P*<0.05.

## Results

***Elevated expression levels of VEGF, HIF-1α, and p-AKT in OA***

**Figure 1:**
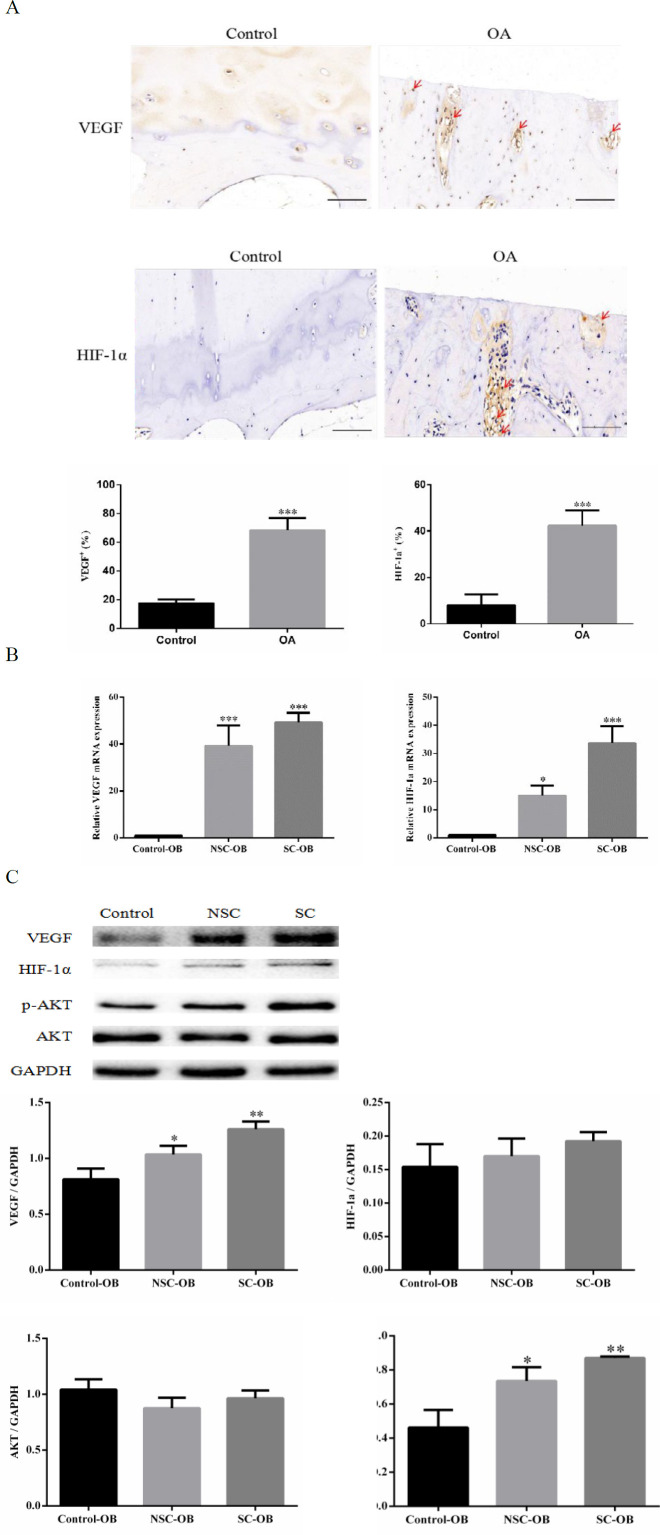
The high expression of VEGF and HIF-1α in OA subchondral bone. (A) Immunohistochemistry of VEGF and HIF-1α (brown) in the subchondral bone between control and OA groups. The quantitative analysis of the number of VEGF+ and HIF-1α+ cells on the right. Scale bars, 100 µm. (B) RT-qPCR analysis of VEGF and HIF-1α in osteoblasts from normal, medial, and lateral subchondral bone. (C) VEGF, HIF-1α, p-AKT, and AKT protein levels in primary osteoblasts and relative quantification for Western blotting. Data are presented as means±SD. *P<0.05, **P<0.01, and ***P<0.001 compared with the control group

Immunohistochemistry confirmed strong staining of VEGF and HIF-1α in OA subchondral bone compared with the control group (Figure 1A). VEGF and HIF-1α mRNA levels in primary SC-OB were higher than those of NSC-OB and control-OB (Figure 1B). Additionally, p-AKT expression in OA osteoblasts was higher, while there was no statistical difference in the protein expression level of HIF-1α and AKT among different groups (Figure 1C). 

***T3 up-regulate the expression of VEGF and HIF-1α by PI3K/AKT signaling pathway***

**Figure 2:**
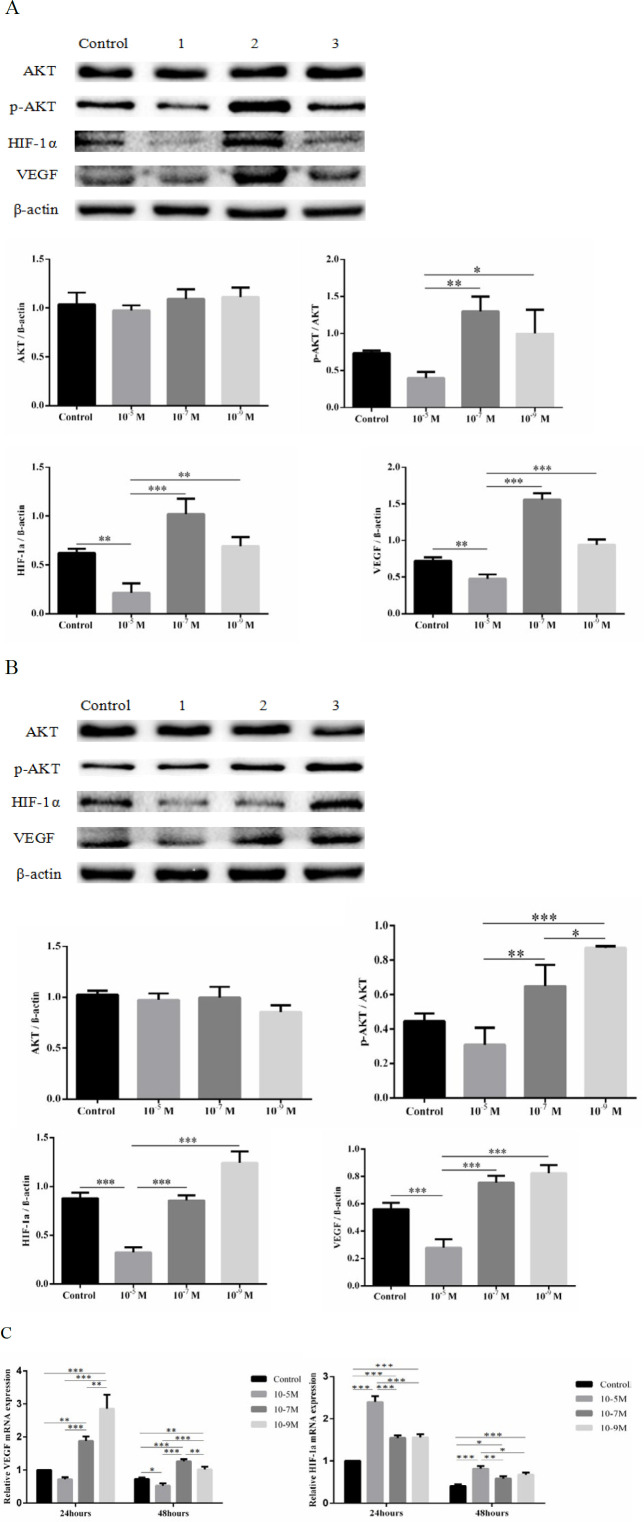
T3 up-regulated the expression of HIF-1α and VEGF. The protein expression and quantification of p-AKT, AKT, HIF-1α, and VEGF after treatment with different doses of T3 for 24 hr (A) or 48 hr (B). Control: no treatment group; Lanes 1, 2, and 3: treatment with T3 at different doses (10^-5^ M, 10^-7^ M, and 10-9 M). (C) VEGF and HIF-1α mRNA expression treated with different doses of T3 for 24 hr and 48 hr. Data are presented as means±SD. *P<0.05, **P<0.01, and ***P<0.001

T3: triiodothyronine; VEGF: vascular endothelial growth factor; HIF-1α: hypoxia-inducible factor-1α; OA: osteoarthritis; AKT: protein kinase B

As shown in Figure 2, the doses of 10^-7^ M and 10^-9^ M T3 increased mRNA and protein expression of VEGF and HIF-1α in OA osteoblasts after 24 hr and 48 hr. However, the dose of 10^-5 ^M T3 reduced the expression of VEGF. Additionally, despite elevated mRNA level of HIF-1α under 10^-5^ M T3 treatment in OA osteoblasts, the protein level was reduced after 24 hr and 48 hr culture. Moreover, the changes in p-AKT protein expression and the ratio of p-AKT/AKT in osteoblasts were consistent with HIF-1α and VEGF (Figures 2A, 2B). 

*Regulative role of HIF-1α in T3- and hypoxia-stimulated VEGF expression*

**Figure 3:**
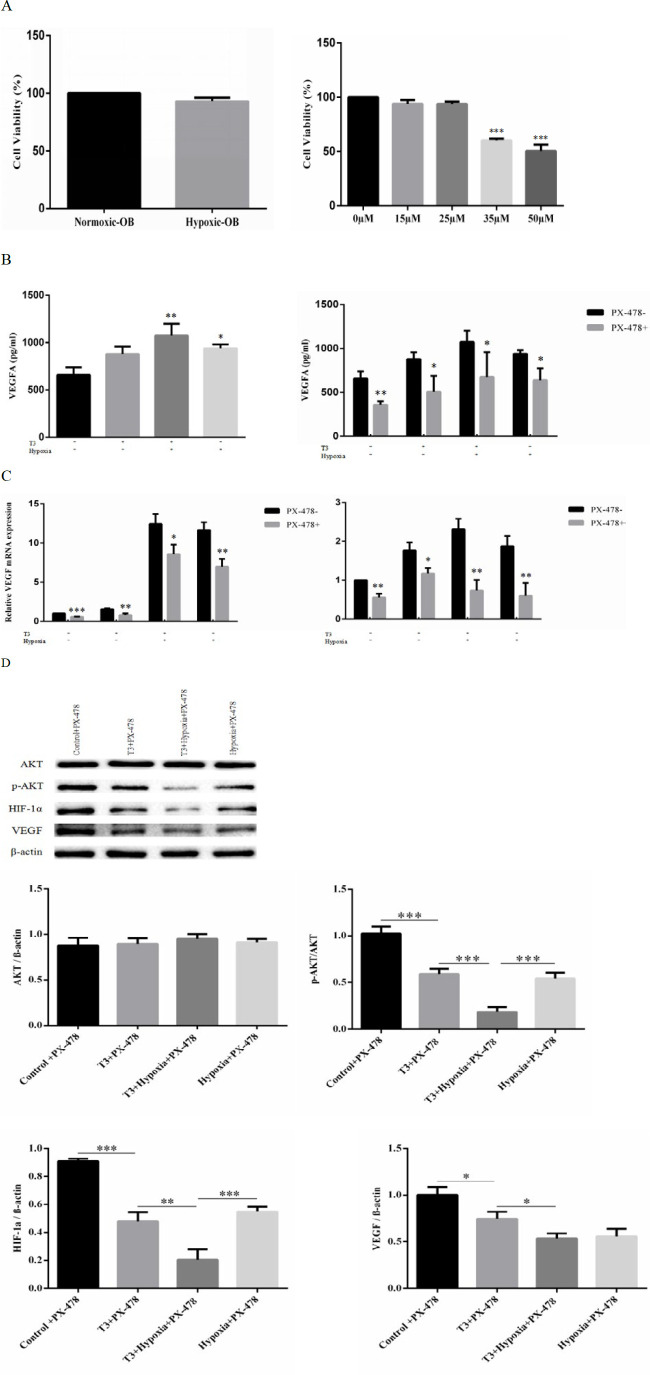
The regulative role of HIF-1α for VEGF expression. (A) Osteoarthritic osteoblasts were treated with hypoxia and different concentrations of PX-478 for 24 hr, and cell viability was detected using a CCK8 assay. (B) VEGF secretion of osteoblasts was detected by ELISA treated with T3 and hypoxia under PX-478 stimulation or without. (C) RT-qPCR analysis of VEGF and HIF-1α treated with and without PX-478 in repose to 10-7M T3 and hypoxia stimulation. (D) p-AKT, AKT, HIF-1α, and VEGF protein levels treated with T3 and hypoxia under PX-478 stimulation and quantification for Western blotting. Data are presented as means±SD. *P<0.05, **P<0.01, and ***P<0.001

VEGF: vascular endothelial growth factor; HIF-1α: hypoxia-inducible factor-1α; AKT: protein kinase B

ELISA detection showed that the level of VEGF secretion was elevated under T3 and hypoxic stimulation. VEGF mRNA level increased 1.6 fold by T3, 11.67 fold by hypoxia, and 12.4 fold by their combination. As the specific inhibitor of HIF-1α, PX-478 was used to identify the mediating role of HIF-1α for VEGF expression in response to T3- and hypoxic-stimulation. The osteoblasts viability was firstly detected treating hypoxia stimulation and different concentrations of PX-478. The data of CCK-8 indicated that hypoxia and the doses of 0 μM, 15 μM, and 25 μM PX-478 did not display any toxic effects on osteoblasts (Figure 3A). ELISA detection demonstrated that the levels of VEGF secretion were obviously increased under T3- and hypoxia-stimulation and this process could be blocked using PX-478 (Figure 3B). Meanwhile, the results of RT-qPCR revealed that PX-478 significantly inhibited T3- and hypoxia-stimulated HIF-1α mRNA levels by 33.7% and 68.1%, respectively (Figure 3C). Consistently, T3- and hypoxia-stimulated HIF-1α protein levels were decreased by 47.8% and 37.8%, respectively in response to PX-478 treatment (Figure 3D). This inhibitor, however, showed an obvious effect on the basal HIF-1α mRNA levels. In addition, for basal protein expression of VEGF, PX-478 also showed significantly inhibiting effect and suppressed T3- and hypoxia-stimulated VEGF production by 48.9% and 41.7% at mRNA level, respectively. 

## Discussion

Angiogenesis in subchondral bone is an important factor of aggravating cartilage degradation, and previous studies have indicated that the pathological changes of subchondral bone may be the initiation of OA (20). In fact, the abnormally proliferating osteoblasts in OA are closely related to subchondral bone sclerosis, inflammation, and angiogenesis. Therefore, in this study, we first examined the expression of VEGF and HIF-1α in OA osteoblasts and found that the expression levels of them were significantly higher. This result proved that, on the one hand, angiogenesis-related factors acted as inducers in the pathological features of OA subchondral bone, and on the other hand, the change of phenotype of NSC-OB, not just SC-OB, also contributed to the OA progression. Consistently, immunohistochemistry of subchondral bone also further confirmed the result. However, unbalanced mechanical stress in OA is closely related to the VEGF over-secretion, and thereby stimulating MMPs expression and cartilage degradation (21). The vascularization of the osteochondral unit is strongly accompanied by pathological changes of subchondral bone by inflammatory cell infiltration, transport of metalloproteinase, and endothelial cell proliferation (22). Additionally, as an important vascular antagonizing cytokine, the decreased activity of CHM-I results in an imbalance between angiogenesis and antagonism (23). Therefore, the application of VEGF antagonists in advanced OA is of great value for the treatment. 

Actually, the rare study reported that T3 could influence OA development and its role was not fully elaborated. T3 also participates in regulating osteoblasts differentiation, bone matrix synthesis, and degradation. Alkaline phosphatase, type I collagen, osteocalcin, MMP9, MMP13, and various growth factors expression can be elevated in response to T3 stimulation in osteoblasts via different intracellular conduction mechanisms (24, 25). As our results showed, VEGF and HIF-1α expression levels were up-regulated under T3 stimulation, primarily the physiological concentration of 10^-7 ^M. However, a higher dose of T3 inhibited the expression of them, representing the consistency with trophoblast cells *in vitro *(26, 27). The weakened angiogenic activities may be attributed to the negative feedback regulation of thyroid hormone receptors to the high concentration of T3 and the osteoblasts death caused by the toxicity of T3. Therefore, possible mechanisms require further exploration in the next study. Thus, the effect of T3 for VEGF and HIF-1α synthesis in OA osteoblasts were dual and local T3 in knee joints played a vital role in OA progression. Additionally, the possible signaling pathway was also investigated in this study. A recent study has also suggested that T3 up-regulated HIF1A and TGFA expression in MCF7 cells by activating the PI3K/AKT signaling pathway (28). This signaling pathway plays a crucial role in regulating angiogenesis, cell growth, differentiation, apoptosis, and inflammation activity (29). As an important protein kinase, the activation of PI3K (phosphatidylinositol 3-kinase) was involved in various growth factors expression and thereby recruiting second messenger PIP3 to conduct the subsequently biological effect by AKT protein phosphorylation (30). Our results found that the changes of p-AKT expression were consistent with VEGF and HIF-1α at protein levels treated with different concentrations of T3. Hence, this finding demonstrated that the PI3K/AKT pathway was required in T3-induced angiogenesis-related factors expression in OA osteoblasts. 

The imbalance of mechanical overload decreases the oxygen supply in the knee joint and induces HIF-1α overexpression (31, 32). HIF-1α acts as a pivotal regulator for oxygen homeostasis. The up-regulation of HIF-1α was also found in our study using RT-qPCR analysis and immunohistochemistry in OA subchondral bone. This result further suggested that oxygen supply was limited in subchondral bone during OA evolution. Conversely, the previous study indicated that the positive effect of HIF-1α was observed in the maintenance of the normal cartilage matrix (33). This finding clarified the dual role of HIF-1α in OA. Meanwhile, previous studies have also confirmed that T3 increased cellular oxygen consumption and led to the hypoxic conditions in the germ cells (34, 35). Taken together, we investigated the effect of T3 and hypoxia treatment for OA osteoblasts and found a higher expression of HIF-1α and VEGF in response to these stimulators. Indeed, as this result showed, PX-478 inhibited HIF-1α activity to down-regulate the expression of VEGF under T3 and hypoxia treatment. Therefore, HIF-1α regulates the expression of VEGF, thereby facilitating angiogenesis. Furthermore, PX-478 showed no markedly inhibitory effects on AKT protein, while significantly decreasing the protein level of p-AKT. Thus, our results have once again identified the regulative role of HIF-1α for T3- and hypoxia-stimulated VEGF secretion in OA osteoblasts, and the process was mediated via the PI3K/AKT signaling pathway.

**Table 1:** Demographic parameters of the osteoarthritis (OA) group and healthy control group

	Age (Year)	Gender	K-L grade
OA	60	Female	Ⅳ
	69	Male	Ⅳ
	72	Female	Ⅳ
	79	Male	Ⅳ
	81	Female	Ⅳ
	63	Female	Ⅳ
	83	Female	Ⅳ
	74	Female	Ⅳ
	71	Female	Ⅳ
	69	Male	Ⅳ
	61	Female	Ⅳ
	62	Female	Ⅳ
Healthy controls	62	Male	0
	60	Female	0
	61	Female	0
	73	Female	0
	78	Male	0

VEGF: vascular endothelial growth factor; HIF-1α: hypoxia-inducible factor-1α; OA: osteoarthritis; AKT: protein kinase B

## Conclusion

In summary, VEGF and HIF-1α expression were up-regulated under T3 stimulation in OA osteoblasts through the PI3K/AKT signaling pathway. Additionally, inhibition of HIF-1α activity could obviously down-regulate VEGF and p-AKT expression, which implied its importance in angiogenesis of OA subchondral bone.
